# Harvesting changes mating behaviour in European lobster

**DOI:** 10.1111/eva.12611

**Published:** 2018-03-22

**Authors:** Tonje K. Sørdalen, Kim T. Halvorsen, Hugo B. Harrison, Charlie D. Ellis, Leif Asbjørn Vøllestad, Halvor Knutsen, Even Moland, Esben M. Olsen

**Affiliations:** ^1^ Department of Biology Centre for Ecological and Evolutionary Synthesis (CEES) University of Oslo Oslo Norway; ^2^ Department of Natural Sciences Centre for Coastal Research (CCR) University of Agder Kristiansand Norway; ^3^ Institute of Marine Research His Norway; ^4^ Australian Research Council Centre of Excellence for Coral Reef Studies James Cook University Townsville QLD Australia; ^5^ National Lobster Hatchery Padstow UK

**Keywords:** assortative mating, *Homarus gammarus*, marine protected areas, mating behaviour, parentage analysis, sexual selection

## Abstract

Removing individuals from a wild population can affect the availability of prospective mates and the outcome of competitive interactions, with subsequent effects on mating patterns and sexual selection. Consequently, the rate of harvest‐induced evolution is predicted to be strongly dependent on the strength and dynamics of sexual selection, yet there is limited empirical knowledge on the interplay between selective harvesting and the mating systems of exploited species. In this study, we used genetic parentage assignment to compare mating patterns of the highly valued and overexploited European lobster (*Homarus gammarus*) in a designated lobster reserve and nearby fished area in southern Norway. In the area open to fishing, the fishery is regulated by a closed season, a minimum legal size and a ban on the harvest of egg‐bearing females. Due to the differences in size and sex‐specific fishing mortality between the two areas, males and females are of approximately equal average size in the fished area, whereas males tend to be larger in the reserve. Our results show that females would mate with males larger than their own body size, but the relative size difference was significantly larger in the reserve. Sexual selection acted positively on both body size and claw size in males in the reserve, while it was nonsignificant in fished areas. This strongly suggests that size truncation of males by fishing reduces the variability of traits that sexual selection acts upon. If fisheries continue to target large individuals (particularly males) with higher relative reproductive success, the weakening of sexual selection will likely accelerate fisheries‐induced evolution towards smaller body size.

## 1 INTRODUCTION

Humans depend on healthy ecosystems for valuable goods and services, but human activities are also considered to be one of the strongest selective forces in nature (Palumbi, 2001). For instance, harvesting is virtually always nonrandom and disproportionally removes certain phenotypes from the population (Allendorf & Hard, 2009; Hutchings & Rowe, 2008). Typically, harvesting targets large individuals due to marked preferences or management regulations imposing minimum‐size limits (Beamish, McFarlane, & Benson, 2006; Berkeley, Chapman, & Sogard, 2004). There is mounting empirical evidence showing that such size‐selective harvesting can drive contemporary evolution of life‐history traits (Enberg et al., 2012; Heino, Dìaz Pauli, & Diekmann, 2015; Uusi‐Heikkilä et al., 2015) with consequences for population productivity and persistence (Jørgensen, Ernande, & Fiksen, 2009; Jørgensen et al., 2007; Kuparinen & Merilä, 2007). However, far less attention has been dedicated to the interaction between human‐induced mortality and mating systems of exploited populations (Fenberg & Roy, 2008; Lane, Forrest, & Willis, 2011; Rowe & Hutchings, 2003). More so, the potential contribution of male phenotype to populations’ reproductive success, and factors underlying variation in the intensity of sexual selection on male traits, remains largely ignored (Uusi‐Heikkilä et al., 2015). This paucity of research is surprising for several reasons. First, harvesting tends to select against sexually selected characters, such as the size of weaponry (e.g., horns, antlers and claws) and body size; traits that are generally important in mate choice and intraspecific competition for access to mates (Swain et al., 2007; Wilber, 1989; Woolmer, Woo, & Bayes, 2013). Coltman et al. (2003) demonstrated this effect in bighorn sheep (*Ovis canadensis*) when the harvest of the larger and sexually dominant males for trophies led to artificial evolution towards smaller horn size and a reduction in male body size (Coltman et al., 2003; Pigeon, Festa‐Bianchet, Coltman, & Pelletier, 2016). Second, if harvesting alters sex ratios (e.g., Kendall & Quinn, 2012), this will likely influence the opportunity and strength of sexual selection (Kokko, Klug, & Jennions, 2012; Kokko & Rankin, 2006). Third, the strength of sexual selection on fecundity and mating success can be stronger than that generated by natural selection (Kingsolver et al., 2001; Siepielski, DiBattista, Evans, & Carlson, 2011), illustrating the necessity of considering sexual selection when predicting evolutionary rates and trajectories of harvested populations.

To the best of our knowledge, parentage assignment techniques have never been used to directly address how harvesting may potentially disrupt natural processes of sexual selection in the marine environment. Hutchings and Rowe's (2008) modelling work on the Atlantic cod (*Gadus morhua*) showed that if reproductive success increases with body size and harvesting decreases its mean and variation, the overall strength of selection for smaller body size is stronger than expected by fishing alone. Disentangling how harvesting might affect the stability of a mating system is no trivial task, especially in many marine species which are not easily observed in their natural environment. Most studies of sexual selection and mate choice have been limited to controlled environments and model species (Rowe, Hutchings, Skjæraasen, & Bezanson, 2008; Uusi‐Heikkilä, 2012), but discrepancy in results between laboratory and field studies underscore the need for more research on mating behaviour in the wild (Lane et al., 2011; Mobley, Abou Chakra, & Jones, 2014). Considering that many commercially fished species are regarded as fully‐ or overexploited (Worm, Hilborn, Baum, & Zeller, 2009), few locations for these species remain where natural mating dynamics are likely to be intact (Fenberg & Roy, 2008; Rowe & Hutchings, 2003). No‐take marine reserves, where population demographic characteristics such as density, sex ratios and size composition are expected to be restored towards baseline conditions (Berkeley et al., 2004; Birkeland & Dayton, 2005), are therefore particularly valuable as reference systems when exploring fisheries effects on mating systems (Butler, Bertelsen, & MacDiarmid, 2015).

**Figure 1:**
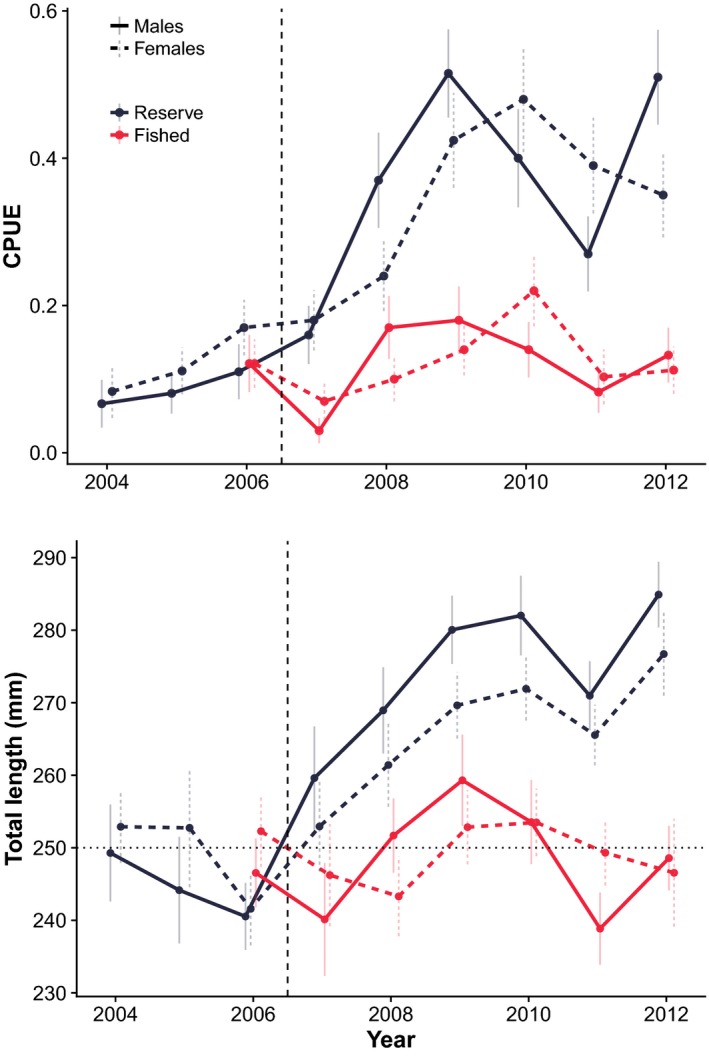
Catch and size distribution of the lobster population in reserve and fished area. Mean catch‐per‐unit‐effort (CPUE) of legal sized European lobster (upper panel) and total body length (mm) from the annual research trap survey prior to establishment of the reserve (2004–2006) and after (2006–2013, indicated by vertical stippled line), with reserve in dark grey and fished area in red colour. The error bars depict standard error around the mean. Sex is separated with males in solid line and females in stippled line. The stippled horizontal line denotes the minimum legal size for lobsters in Norway (25 cm)

We investigated potential effects of harvesting on the mating system of the European lobster (*Homarus gammarus*) by comparing paternity data from a lobster reserve and an adjacent area open to fishing across multiple years. The clawed lobsters, consisting of European lobster and the American lobster (*Homarus americanus*), are long‐lived iconic species with high commercial value and therefore subject to intense fishing pressure (Anonymous 1995; Kleiven, Olsen, & Vølstad, 2012). The Norwegian lobster fishery is regulated by closed season, minimum legal size (>250 mm total length, TL) and a ban on the harvest of egg‐bearing females (since 2008). Laboratory studies show that when a female is ready to mate, she will seek out a male and preferentially choose a large individual as mate (Bushmann & Atema, 1997; Karnofsky, Atema, & Elgin, 1989a,b; Skog, 2009a). Given that sperm limitation may occur in many crustacean species (Hines et al., 2003; Jivoff, 2003; Kendall & Wolcott, 1999; Kendall, Wolcott, Wolcott, & Hines, 2002; MacDiarmid, Butler, & Butler, 1999; Sato, Ashidate, Jinbo, & Goshima, 2006), females would expectedly prefer to mate with males of similar or larger size to ensure passing of sufficient sperm. In addition, males should also favour large females as egg production increases exponentially with increasing female size (Wahle, Castro, Tully, & Cobb, 2013). Our first objective was to determine to what extent there is a consistent relative size difference between mated pairs in the two areas and whether size‐assortative mating—the nonrandom association of body size between mated individuals—exists. Probably because of the disparate conservation regulations between the areas (and sexes due to mandatory return of egg‐bearing females in the fished area), the mean size differences between males and females are smaller in the fished area relative to the reserve (Figure 1). We therefore predicted that females should mate with males of smaller sizes in the fished area compared to females in the reserve, thus creating a weaker pattern of size‐assortative mating.

Body size has been shown to be under sexual selection in many crustaceans (e.g., Bertin & Cézilly, 2003; Karnofsky & Price, 1989). In clawed lobsters, male–male competition is intense and males fight over shelters and contest dominance (Atema, 1986; Skog, 2009b). Males have relatively larger claws than females (Debuse, Addison, & Reynolds, 2001; Templeman, 1935, 1944) and larger claws increase a male's competitive abilities (Atema & Cobb, 1980; Elner & Campbell, 1981), so claw size should therefore be under strong sexual selection. Thus, our second objective was to estimate and compare the strength of sexual selection, within a breeding season, on two male traits: body size (carapace length, CL) and absolute and relative claw size (width of crusher claws, CW). Aligning with our hypothesis of weaker size‐assortative mating in the fished area, we predict that selection differentials, that is, the difference in these mean trait values between successful and unsuccessful males, to be larger in the reserve than the fished area because of the reduced trait variability in the fished area.

Our results contribute to a broader understanding of fisheries‐induced evolution by quantifying fisheries‐induced changes to mating systems and sexual selection, relevant for developing management tools aimed at mitigating long‐term negative impact of selective harvesting. Specifically, we argue that fisheries targeting large males with high reproductive success can lead to a weakening of sexual selection which could further accelerate fisheries‐induced evolution towards less productive (smaller) phenotypes.

## 2 MATERIALS AND METHODS

### 2.1 Study system and lobster sampling

**Figure 2:**
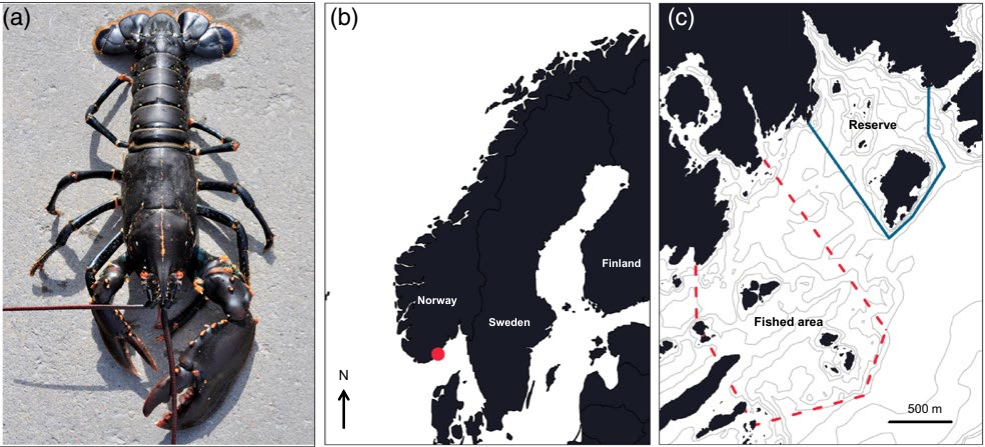
Sampling location. Study species, European lobster (*Homarus gammarus*) (a), study area on the Norwegian Skagerrak coast (circular marking) (b) and Flødevigen lobster reserve and fished area (c). Solid line: reserve boundary, stippled line: monitored fished area.

The study was conducted in an area open to fishing and in a designated lobster reserve established in September of 2006, located at the Skagerrak coast in south‐eastern Norway (Figure 2). The reserve and the monitored fished area are separated by a distance of ~800 m, and mark–recapture data suggest very little exchange of individuals between the two areas (Thorbjørnsen, 2015). Temporal trends in catch‐per‐unit‐effort and length data from a standardized research trapping survey are presented in Figure 1. Briefly, this annual survey samples lobsters using standard parlour traps set at 5–30 m depth during 4 days in late August/early September. The reserve and fished area are fished with the same effort (100 hauls per year), see Moland et al. (2013) for details. Of the egg‐bearing females sampled, 108 were caught from June to September in 2011 and 2012 (60 from the reserve and 48 from the fished area). Because more lobsters are caught in the reserve, seven additional females were obtained from the fished area with help from local fishermen during the ordinary fishing season in October–December 2012 to achieve a balanced sample size in the two areas. Males were fished extensively throughout 2010–2013 from June to December in order to include as many paternal candidates as possible in the parentage analysis. Most of the males were sampled as part of the standardized research trapping survey described above. Additionally, males were sampled when fishing for females in 2011 and 2012 and in conjunction with another study in the fished area in 2011 (Wiig, Moland, Haugen, & Olsen, 2013). Captured lobsters were sexed, measured and individually tagged with externally visible T‐bar tags (TBA2, 45 × 2 mm, Hallprint). Claw width and carapace length (CL—rear of the eye socket to the rear of the carapace) was measured to nearest millimetre. A small piece of tissue from the tip of the foremost pleopod was stored in pure ethanol for later genetic profiling. All lobsters were released at the sampling site. Where males were recaptured in successive years, the freshest tissue sample was genotyped to ensure the highest DNA quality.

### 2.2 Genetic sampling of female and offspring

Captured egg‐bearing females were also measured and individually tagged with T‐bar tags. In addition, tissue samples were collected along with samples of offspring, where one egg was randomly sampled at the top of the egg mass near each of the ten pleopods and stored in separate vials with ethanol (a total of ten vials with eggs from each female). Sampling fertilized eggs of each female allowed us to deduce the genotype of the father of each offspring based on the known mother–offspring genotype combination, which should help increase the likelihood of finding the actual fathers when running parentage assignment with the sampled males.

### 2.3 DNA extraction and microsatellite genotyping

DNA was extracted from 60 females from the reserve area and 55 females from the fished area, all 650 males sampled (*n*_reserve_ = 331, *n*_fished_ = 319), and a total of 1,150 offspring from the 115 females. All individuals were genotyped with ten microsatellite loci developed for European lobster (see André and Knutsen 2009 for primer sequences). The DNA was extracted with E.Z.N.A. Tissue DNA Isolation kit (Omega Bio‐Tec inc.) and PCR product amplified on MyCycler ^™^ (Bio‐Rad) using fluorescent‐dyed forward primers (Life Technologies). The loci were pooled into one triplex (loci *hgd* *106,* *hgd* *111* and *hgc* *118*), three duplexes (loci *hgc* *111* and *hgc* *131,* *hgc* *129* and *hga* *8,* *hgb* *4* and *hgb* *6*) and one simplex (locus *hgc* *120*). Fragment analysis of PCR products was carried out on capillary sequencers CEQ^™^8000 (Beckman Coulter) and ABI ^™^ 3130xl (Applied Biosystems) and manually scored using genemapper v3.7 (Applied Biosystems) and ceq ^™^ 8000 genetic analysis system v 8.0, respectively. As the length of the alleles slightly differed between the instruments, msatallele (Alberto, 2009), a script build on R, was used to bin the scored raw sizes from both fragment analysers and correctly calibrate the results from the two. To control cross‐contamination of samples, a negative control was included in each 96‐well plates used for PCR and electrophoreses. All candidate males with assigned parentage in the initial analysis were re‐extracted and re‐amplified to rule out errors. The assigned genotypes were also checked manually three times to minimize scoring errors. Genotypes that could not reliably be solved after three repeat‐runs were left as missing. Individuals for which genotypes were missing at five or more loci were considered of poor DNA quality and excluded from further analysis. See Appendix S1.1 for additional details.

### 2.4 Genetic analysis

The identity check function in cervus v. 3.0.3 (Kalinowski, Taper, & Marshall, 2007; Marshall, Slate, Kruuk, & Pemberton, 1998) enabled us to identify and remove duplicate samples by checking for identical genotype entries. Such duplicates may be due to tag‐loss and thus repeated tagging. cervus identified 38 duplicated genotypes among the 650 sampled males. When size and expected annual growth were compared in the recapture data for the 38 males, tag‐loss was confirmed as the most probable cause in all cases, and these duplicates were subsequently removed from the candidate file.

Genetic variation within samples was estimated for the adult samples only. We estimated the genetic differentiation between the lobster sampled in the reserve and the fished area using Wright's *F*_ST_, with Weir and Cockerham's (1984) estimator, θ, in genepop v. 4.5. Allele frequency heterogeneity between localities (years pooled) was tested using fstat v.2.9.3.2 (Goudet, 1995). As there was no significant genetic differentiation between the two areas (*F*_ST_ from 0.000 to 0.002, all *p* > .99), all samples were pooled in subsequent analysis. The fixation index (smallF, *F*_IS_) was measured at each locus with fstat. One sample *t* test was used to assess whether *F*_IS_ estimates differed significantly from zero. Pairwise linkage disequilibrium for each pair of loci was tested with a likelihood ratio statistic using the Markov chain algorithm of Raymond and Rousset (1995) in genepop v. 4.5 as well as deviations from Hardy–Weinberg equilibrium (HWE, exact test). All critical significance levels for multiple testing were adjusted with R‐package fdrtool, after Benjamini and Hochberg (1995). Finally, genetic diversity estimates including allele number at each locus (*N*_A_) and the theoretical exclusion probability given one parent is known for each locus and combined (EXC) were estimated with gerud 2 (Jones, 2005). Locus‐specific genotyping error rates, allelic drop‐out (ε_1_) and false allele (ε_2_), were estimated with a combination of methods (see Table 1 for error rates). For details on descriptive population genetics, and approach and estimation of error rates, see Appendix (S1.2–4).

**Table 1:** Description of loci used in the paternity analysis and error rates

Locus	*N*_*a*_	*H*_O_	*H*_E_	EXP	Uncorrected *p*‐value	*F*_IS_	*F* (null)	ε_1_ a	ε_2_ b
*C118*	9	0.619	0.587	0.370	.073	−0.060	−0.031	0.013	*0.010*
*D106*	9	0.703	0.709	0.494	.431	0.013	0.012	0.012^m^	*0.010*
*D111*	12	0.645	0.631	0.405	.909	−0.024	−0.012	0.000	*0.010*
*C131*	13	0.806	0.830	0.669	.231	0.025	0.012	0.012^m^	0.023
*C120*	19	0.844	0.870	0.745	.001	0.009	0.008	0.023	0.013
*C111*	9	0.725	0.735	0.529	.017	0.021	0.001	0.001^m^	0.018
*A8*	14	0.712	0.818	0.661	.000	0.116	0.062*	0.062^m^	*0.010*
*B4*	9	0.606	0.606	0.399	.000	−0.004	0.001	0.006	0.010
*B6*	11	0.738	0.818	0.646	.000	0.001	0.044*	0.044^m^	*0.010*
*C129*	14	0.706	0.779	0.582	.000	0.096	0.040*	0.040^m^	*0.010*
*Average*	11.9	0.710	0.738	0.999		0.023	0.014	0.021	0.012

Number of alleles *N*_*a*_ and observed (*H*_O_) and expected (*H*_E_) microsatellite heterozygosity for the adult European lobster at Flødevigen area, south‐east Norway in 2010–2013. Also given are the expected exclusion probabilities (EXP) of the second parent: the probability of excluding a randomly chosen nonfather when the mother is known, critical *p*‐value for HWE test (*a* = 0.05); *F*_IS_, inbreeding coefficient; *F*(null), loci denoted “*”showing null alleles at high frequency, frequency of null alleles; ε_1_, allelic drop‐out rate; ε_2_, false allele rate. The samples are based on 727 (612 males and 115 female) lobsters. EXP and *average* EXP calculated by GERUD2 according to the equations in Dodds et al. (1996).

ε_1_^a^ = Allelic drop‐out rate estimated from Pedant and Micro‐checker, the latter is denoted “^m^.”

ε_2_^b^ = False allele rate estimated from Pedant. Where Pedant estimated 0.000, 0.010 was implemented in COLONY2, shown in italic.

### 2.5 Paternity and multiple mating analyses

Genotypes from seven males were excluded due to missing data (≥5 missing loci), and eighteen females had eggs from which DNA yield was insufficient to allow successful genotyping of the batches. Altogether, a total of 612 males, 97 females (*n*_reserve_ = 51, *n*_fished_ = 46) and 967 eggs were used (and pooled) in the final parentage analysis (Table 2). We assigned parentage using colony v 2.0 (Jones & Wang, 2010; Wang, 2004), a full‐pedigree likelihood program (Markov chain Monte Carlo method) that provides the most probable configuration in assigning sib‐ship and parentage among individuals. We allowed both females and males to be polygamous, a prerequisite for testing multiple paternities in regard to both sexes. We accepted only paternities assigned with 95% confidence or higher. This helped minimize false‐positive and false‐negative assignments and avoided overestimating the level of multiple paternity in the population. Although not all fathers were sampled, colony can infer their genotypes from the pedigree analysis to the number of mates to each female and infers the most likely number of fathers contributing to each batch. Where colony inferred more than one sire in a batch, visual inspection of genotypes and changes made by colony based on the error rates, helped minimize an overestimation of multiple paternity cases (due to for example contamination from mother's DNA, multiple reconstructions of alleles suggested with almost equal probabilities). Inferred multiple paternities were only accepted as true cases of multiple paternity if offspring differed from the first male at five or more loci, did not show sign of scoring error (miss‐matching mothers genotype) and if the loci in question had not been calculated by colony due to missing alleles. The input files in colony were set up with two replicate runs and analysed with the highest precision settings with full‐likelihood, and with very long runtime on a PowerEdge M820, Linux CentOS 6.7 machine. For more details on the settings used, see Appendix (S1.3).

**Table 2:** Summary results on European lobsters used in the analysis separated in year and area

Area	2010		2011		2012		Years pooled	
	Reserve	Fished	Reserve	Fished	Reserve	Fished	Reserve	Fished
Females								
No. females (No. of offspring)	–	–	42 (420)	27 (269)	9 (90)	19 (188)	51 (510)	46 (457)
Mean carapace length (CV), mm	–	–	96 (0.11)	91 (0.10)	105 (0.15)	94 (0.13)	97 (0.12)	92 (0.11)
No. offspring assigned candidate male	–	–	296 (70%)	89 (33%)	57 (63%)	69 (36%)	353 (69%)	158 (34%)
Males								
No. males (No. of candidate assigned)	98 (20)	80 (5)	148 (11)	111 (8)	28 (5)	96 (5)	274 (36)	287 (18)
Mean carapace length (CV), mm	104 (0.18)	90 (0.13)	95 (0.17)	88 (0.15)	101 (0.19)	88 (0.16)	99 (0.18)	88 (0.15)
Mean claw width (CV), mm	58 (0.25)	46 (0.18)	51 (0.24)	44 (0.21)	55 (0.32)	44 (0.22)	54 (0.26)	45 (0.20)
St. Selection diff* carapace (*p*‐value)	**0.80** (<0.01)	−0.37 (0.53)	**0.45** (0.01)	0.29 (0.33)	**1.66** (0.06)	0.43 (0.37)	**0.78** (<0.01)	0.16 (0.48)
St. Selection diff* claw width (*p*‐value)	**0.94** (<0.01)	−0.25 (0.67)	**0.67** (<0.01)	0.22 (0.45)	**1.66** (0.08)	0.7 (0.19)	**0.93** (<0.01)	0.22 (0.34)

For females, number of females and number of offspring in parentheses, mean carapace length in mm with corresponding coefficient of variation (CV), the number and percentage of offspring assigned candidate males. For males, number of candidates and assigned males in parentheses, mean carapace length and crusher claw width in millimetres with corresponding coefficient of variation (CV), standardized selection differentials (diff*) for body size and claw width with confidence value (*p*‐value) in parentheses. Significant selection differentials are in bold. Only paternity assigned at 95% confidence is reported and counts the number of matings by known males, including males that have mated with multiple females and hence appear more than once in the counts.

The probabilities of detecting multiple paternal contribution (prDM) were quantified using the software prDM (Neff, Pitcher, & Repka, 2002). prDM uses Monte Carlo simulations to calculate prDM under various scenarios of skew between the fertilization contributions of multiple males based on population allele frequencies. When determining the frequency of multiple sired batches, we inspected the results that were flagged as cases of multiple paternity by colony along with the original genotype data. This is because colony can alter loci in accordance with error estimates and propose alleles in cases where genotypes are missing and that could overestimate multiple paternity cases. The offspring batches would only be resolved as cases of multiple paternity if the genotype of an offspring could not be resolved by the first male by at least five loci, did not show evidence of contamination/amplification issue or had loci altered by colony.

### 2.6 Size‐assortative mating

We first compared the overall size of females and males in the whole data set in both areas with a two‐tailed *t* test. For analysing the size relationship between mated pairs, we had to account for the fact that lobsters were captured across several mating seasons. The majority of females have a biennial reproductive cycle, whereby spermatophores received during mating are stored for 9–11 months and used to externally fertilize eggs prior to incubation for a similar duration, after which they moult and remate (Agnalt, Kristiansen, & Jørstad, 2007; Aiken, Mercer, & Waddy, 2004). Thus, the egg‐bearing females with newly extruded (black) eggs sampled in 2011 and 2012 most likely mated some time in 2010 and 2011. The sampling year of inferred fathers differed from the sampling year of the female in most of the mated pairs (70%). For these pairs, it was necessary to estimate male size for the time at which corresponding females were sampled prior to analysing size‐assortative mating patterns. To this end, we used mark–recapture data from the reserve and fished area from 2004‐2016 and extracted males that had been captured in two consecutive years at any point within this period. First, we estimated the probability of moulting as a function of carapace length at the first capture with a logistic regression. We inferred that moulting had occurred if the size difference was 5 mm or higher from the previous year; smaller differences were assumed to be measurement errors (Agnalt et al., 2007). We then estimated the yearly growth increment as a function of carapace length at the time of first capture for individuals who had moulted, using a linear regression. The predicted values from these two models were included in the calculation of adjusted carapace length for males with mating success using the following formula:

(1) $${\text{CL}}_{\mathrm{adjusted}}={\text{CL}}_{\mathrm{measured}}+\left({\text{Year}}_{f}-{\text{Year}}_{m}\right)\times \hat{g}\times {\hat{p}}_{\mathrm{moult}}$$

where CL is the male carapace length (mm), Year is the year of sampling for males (m) and females (f), and $\hat{g}$ and ${\hat{p}}_{\mathrm{moult}}$ are the estimated yearly growth increment (in mm) and probability of moulting, respectively, as predicted from CL_measured_ using linear and logistic regression. The model predictions showed that almost all males below 90 mm CL (the minimum legal size) moulted annually and that the probability of moulting decreases to below 0.75 in larger sizes classes (>113 mm CL). The overall probability that males of all sizes would moult once every year was >0.5 (see Appendix S2, Figure S1).

With the adjusted male sizes, we used a linear model to test for assortative mating (a positive correlation between female and male body size in mated pairs) and tested whether such patterns differed between areas, comparing models with Area x female size – interaction (Equation (2)) against a model with only an additive area effect with the likelihood ratio test.

(2) $${\text{CL}}_{\mathrm{male}}={\text{CL}}_{\mathrm{female}}+\text{Area}+{\text{CL}}_{\mathrm{female}}\times \text{Area}$$

We excluded six putative matings between mates sampled in different areas. Males were duplicated in the data file if the same male has mated with multiple females.

### 2.7 Selection on male traits

Standardized selection differentials on male body size and claw size were calculated, subtracting the mean trait value of potential fathers from the mean of successful fathers in each area (Arnold and Wade 1984). The size of maturity for males is not known in this population, but to reduce the probability for including immature males among potential fathers, only males with 80 mm or larger CL were included in the selection differential calculations. Size at maturity for European lobster in Scotland has been estimated to 80 mm CL for males and 79 CL for females (Lizárraga‐Cubedo, Tuck, Bailey, Pierce, & Kinnear, 2003). However, the smallest berried female in our sampling was 73 mm CL, compared to 82 mm in the Scottish study; thus, we consider a potential father threshold of 80 mm CL and above to be conservative and appropriate for our study system. Prior to calculations, trait values were mean‐centred and scaled to a standard deviation of one in each area–year combination (Lande & Arnold, 1983). Significance of selection differentials was assessed with two‐tailed t tests. Also, a linear model was used to compare the body size (CL) of males that were successfully assigned and, thus successfully sire offspring, to males that had not.

(3) $${\text{CL}}_{\mathrm{male}}=\text{Assigned}+\text{Area}+\text{Year}+\text{Assigned}\times \text{Area}$$

Of interest was whether the difference in mean trait value between successful and unsuccessful males would be larger in the reserve (a significant interaction effect between area and assignment). Year was included as an additive effect in the model to account for variable trait distribution among sampling years. To test whether the proportion of males assigned differed between areas, we used univariate generalized linear models for each year (2010–2012), where Assigned (0, 1) was the binomial response variable and Area the predictor.

Because males were sampled over three seasons and females in two, the aforementioned selection differentials may not reflect pure sexual selection, as mortality (both fishing and natural) would evidently determine the prospects of obtaining mating success in the different years. Although we maintain that sexual selection is likely to be the primary mechanism underlying these selection differentials, we also conducted a more specific analysis of sexual selection, where we included only males sampled in 2010 because they represent the population at the time of reproduction and also had a sufficient number of assigned paternities (in following year) to warrant further analyses (2010: 24 out of 245; 2011: 5 out of 272). We estimated standardized selection gradients, which capture the sensitivity in the fitness function when trait values change, and therefore better represent the strength and shape of selection than selection differentials alone (Kingsolver, Diamond, Siepielski, & Carlson, 2012; Matsumura, Arlinghaus, & Dieckmann, 2011). Selection gradients were estimated from logistic regressions (Janzen & Stern, 1998) on male body size (CL) and claw size (CW), with mating success (*s*) as the response variable (0 or 1). Claw width and body size were strongly correlated traits (*r* = .90) and could therefore not be included in the same model due to high collinearity (Lande & Arnold, 1983; Zuur, Ieno, & Elphick, 2010). Thus, to include both traits, we extracted the residuals from the linear regression between claw width and carapace length and used the residual claw size, which is then a measure of relative claw size (CW_res_), as covariate together with CL. We also fitted a model only including relative claw size (CW_res_). To evaluate whether trait‐fitness relationships differed between areas, we also included models testing for an interaction effect with area for each of the traits (CL, CW and CW_res_) using the following model structures:

(4) logit(s)=CL+Area+CL×Area

(5) logit(s)=CW+Area+CW×Area

(6) $$\text{logit}\left(s\right)=\text{CL}+{\text{CW}}_{\mathrm{res}}+\text{Area}+{\text{CW}}_{\mathrm{res}}\times \text{Area}$$

We also explored whether the data supported stabilizing (i.e., nonlinear), rather than directional (i.e., linear) selection on male size, as recent studies have shown that male mating success might be highest for intermediate sized males (Uusi‐Heikkilä, Kuparinen, Wolter, Meinelt, & Arlinghaus, 2012). For this, we ran models including a squared term for absolute size (body or claw), exemplified for CL below:

(7) $$\text{logit}\left(s\right)=\text{CL}+{\text{CL}}^{2}$$

All selection gradient models (full and reduced) were compared with the Akaike information criterion, corrected for small sample size, which was used to determine the most parsimonious model. We estimated approximate selection gradients (β_avggrad_) for each trait with the Janzen‐Stern logistic regression approach (Janzen & Stern, 1998). Mean standardized selection gradients on claw and body size were calculated by multiplying β_avggrad_ by the trait value′s mean and dividing by its standard deviation (Matsumura et al., 2011). The mean standardized selection gradient is recommended for comparing strength of selection across studies but is not applicable for trait such as relative claw size, which has no natural maximum and minimum value (Hereford, Hansen, & Houle, 2004 and Matsumura et al., 2011). All statistical analyses were performed in R 3.2.4 (R Core Team, 2016).

## 3 RESULTS

### 3.1 Lobster samples and population genetics

The proportion of loci typed over all individuals was 0.946 (adults and offspring; 0.983 and 0.934 respectively, see Appendix S2, Table S1 for females and eggs analysed) and the genetic diversity was high across all loci (*H*_E_ = 0.738; Table 1). The number of alleles per locus ranged from 9 to 19, and the observed heterozygosity ranged from 0.606 to 0.844. We estimated the combined exclusion probability to be .9998 given a known maternal genotype, indicating sufficient power to distinguish between two randomly selected candidate males (though the effect of error rates is not accounted for in the estimation, equation from Dodds, Tate, McEwan, & Crawford, 1996). No parentage was assigned to males sampled in 2013, so all males from this year were removed from further analysis, reducing the number of males to 561 (Table 2). See Appendix (S1.3–4) for additional details.

### 3.2 Mating patterns

A total of 511 (52.8%) offspring were assigned a known father (Table 2). We assigned eggs from 54 females to one of 43 males (7.7% of the 561 candidate males) with high confidence. Of those 54 known matings, 36 (66.7%) involved 27 males from the reserve. Assignment probability differed between the areas in 2010, assigning 19.2% of the (total number of) matings to males in the reserve and 6.4% to males in the fished area (GLM: β *= *1.376, *t* *=* 2.384, *df* *=* 154, *p* *=* .017). colony inferred genotypes from 41 unsampled males that sired offspring with 42 females. There was little exchange of individuals across area boundaries, although five females (reserve *=* 3, fished *=* 2) had mated with males from the opposite area. Two of these interarea pairs involved a large male from the reserve, estimated to have been ~140 mm CL at the time of mating.

Colony initially flagged 24 of the broods to be cases of multiple matings, but after inspecting the assignment results, we concluded that most of the broods probably were sired by one male only because of lack in support of a second sire. However, two (2.0%) of the broods showed evidence of being sired by a second male and therefore concluded to be multiply mated females. The paternal contribution among the multiply mated females was highly skewed in favour of a primary male (9:1 ratio) in both these cases (see Appendix S2 in supplementary information, Table S1). The power to detect multiple paternity with only ten offspring genotyped at ten loci exceeded >99% confidence assuming equal contribution. We could also detect a skew down to 70:20:30 (three sires) with a confidence of more than 95% using nine offspring; however, the skew in favour of a primary male observed in the results (9:1) could only be detected with a 65% confidence. This suggests that, in addition to the two confirmed cases of multiply mated females, some of our single mated females may in fact also be multiply mated.

Of the inferred males with known identity, eight (reserve *=* 6, fished *=* 2) had mated with more than one female, of which five had mated with two females and three had mated with three females. Polygamous males were not significantly larger than males with only one recorded paternity (GLM: β *= *5.956, *t* *=* 1.333, *df* *=* 53, *p* *=* .188). On average, the level of polygamy was higher for males, with females mating with 1.01 males and males (known and unknown) mating with 1.16 females.

### 3.3 Size‐assortative mating

**Figure 3:**
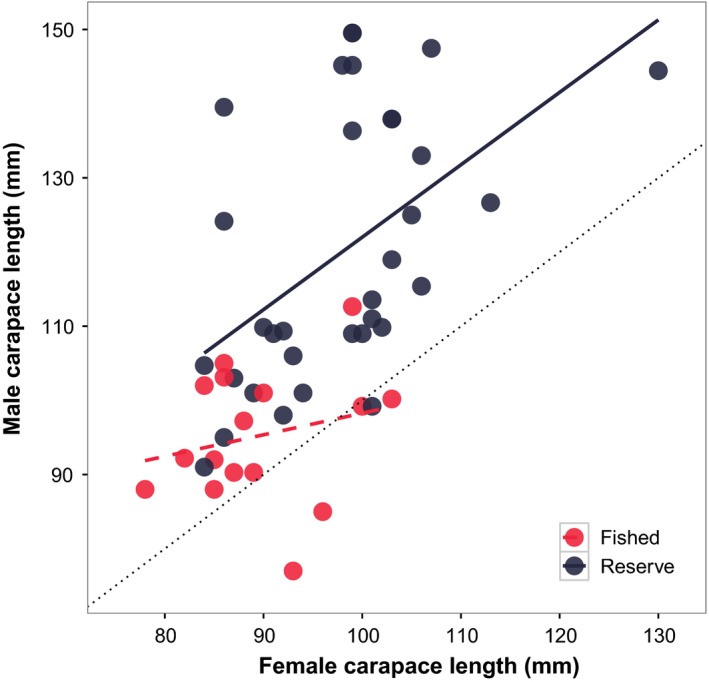
Size‐assortative mating. The relationship between body size (carapace length) of male (corrected sizes, see Materials and methods) and female European lobster that formed pairs (*n* = 51) in fished (red) and reserve (dark grey) area in the four‐year period. Interarea pairs are excluded. Area represents female capture point and male CL is adjusted according to the year of the mating event. Value 1.0 and black stippled line (isometry, *Y* = *X*) marks where females and males are equal in size

Across all sampling years, females were larger than males in the fished area (Table 2; *t* test: *t* *=* 2.12, *df* *=* 68.6, *p*‐value *=* .037), but not in the reserve (Table 2; *t* test: *t* *=* −0.57, *df* *=* 98.5, *p*‐value *=* .57). Interarea pairs (*n* *=* 5) were removed prior to analysing the area‐specific size‐assortative mating pattern (see Appendix S2 in supplementary information, Figure S2). In the reserve, all but two pairs (2 out of 34) consisted of a larger male mating with a smaller female, with an average size difference of 22.5% (*t* test, *t* *=* 6.1799, *df* *=* 48.27, *p* < .0001). Females in the fished area also paired with males of larger sizes, as all but three of the 15 pairs had a male larger than the female, with the average size difference smaller (6.4%) and marginally statistically significant (*t* test: *t* *=* 2.034, *df* *=* 28.35, *p* *=* .051). There was a strong positive size‐assortative mating pattern (GLM: β *= 0.838, t* *=* 3.560, *df* *=* 46, *p* = .0009, multiple *R* ^2^ *=* .50, Figure 3). An additive area effect was supported over an interaction effect (LRT; χ^2^ *=* 1.479, *p* *=* 0.224), with females mating with larger males relative to their own size in the reserve compared to the fished area (GLM: Area: β *=* 17.65, *t* *=* 3.722, *p* *=* .0005).

### 3.4 Sexual selection

**Figure 4:**
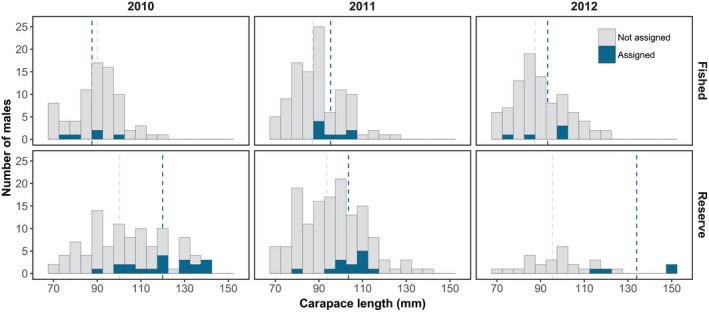
Males with parentage. Length distributions (carapace length, CL in mm) of male European lobsters with (blue) and without (light grey) confirmed assignment in the four sampling years. Vertical lines indicate mean lengths in each group

**Figure 5:**
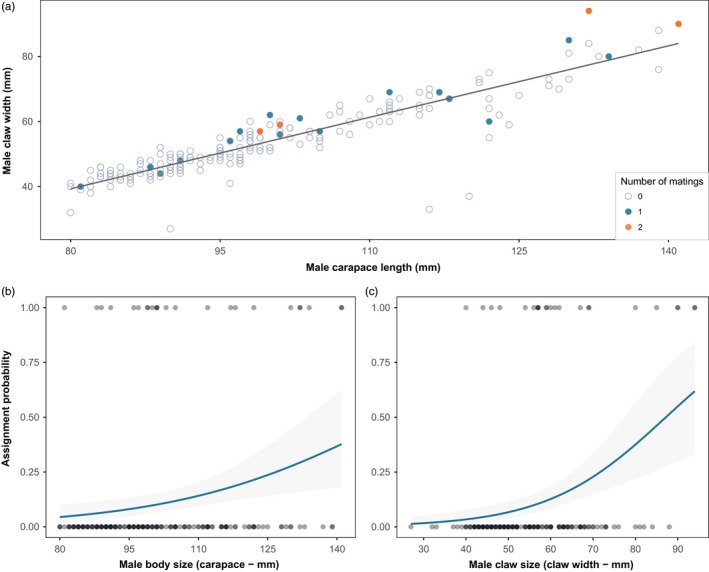
Sexual selection on traits in male European lobsters. (a) Correlation between male carapace length and claw width among males sampled in 2010. Filled coloured circles are showing number of matings (0, 1 and 2) for each male. These residuals were used to estimate sexual selection on relative claw size (Table 4). (b) Probability of mating success as a function of body size among 2010 males (Model 5, Table 3), and (c) probability of mating success as a function of claw size among 2010 males (Model 8, Table 3)

Across all sampling years, selection differentials on body size (CL) and claw size (CW) were significantly positive in the reserve, while they were more variable and nonsignificant in the fished area (Table 2). Correspondingly, the standardized trait difference between successful and unsuccessful males was larger in the reserve for both body size and claw size (GLM: parentage × Area; CL: β *= *0.76, *t* *=* 2.44, *p* *=* .02 and CW: β *=* 0.88, *t* *=* 2.85, *p* *=* .005, Figure 4). The subset of data (2010 mating season) used for estimating sexual selection gradients did not support an area effect on mating success (Table 3). Instead, a model containing only additive effects of body size and residual claw size on male mating success had the lowest AIC_c_ score and therefore the most support (Table 3). A simpler model excluding the effect of body size also received some support (Table 3). Using the most parsimonious model for inference, sexual selection was positive on body size and strongly positive on residual claw size (Table 4). For comparison, univariate selection gradients were significantly positive on all three traits and supported over more complex models including a squared term representing stabilizing or disruptive sexual selection (Table 4, Figure 5).

**Table 3:** Model selection

Model number	Structure	P	AICc
1	CL + CW_res_	3	**128.60**
2	CW_res_	2	129.98
3	CL + CW_res_ × Area	5	132.26
4	CW_res_ × Area	4	134.06
5	CW	2	135.24
6	CW × Area	4	138.18
7	CL	2	143.97
8	CL × Area	4	147.65
9	Null	1	151.04

Logistic regression modelling on selection of male European lobster from 2010 using reproductive success as the response variable. P, number of parameters; AIC, Akaike information criterion score. Explanatory variables (standardized): CL, carapace length; CW, claw width; area, reserve and fished; CW_res,_ relative claw size (residuals from claw body size regression). The model with lowest AIC is indicated in bold.

**Table 4:** Sexual selection estimates

Model no.	Trait	β	*SE*	*z*‐value	*p*	β_avggrad_	β_μ_
1	CW_res_	1.320	0.394	3.350	<.0001	0.965	–
	CL	0.424	0.227	1.868	.06	0.310	–
2	CL	0.609	0.201	3.033	.002	0.512	3.555
3	CW	0.835	0.203	4.111	<.0001	0.657	3.039
4	CW_res_	1.544	0.391	3.949	<.0001	1.170	–

Sexual selection operating on body size and relative claw size in male European lobster sampled in Flødevigen during 2010. For each trait, the table gives Janzen‐Stern logistic regression coefficients (β) and their corresponding standard error (*SE*), *z*‐ and *p*‐value, the approximate selection gradients (β_avggrad_) and the mean standardized selection gradient (β_**μ**_). Traits of interest are carapace length (CL), claw width (CW) and residual claw width (CW_res_), where residuals from the linear regression between carapace length and claw width are used as a proxy for claw size relative to body size. All traits were scaled to a standard deviation of 1 and mean‐centred prior to analysis.

## 4 DISCUSSION

We investigated the mating system of the exploited European lobster in its natural environment inside and outside a coastal marine reserve to establish whether harvesting can affect mating patterns and sexual selection. Our genetic parentage assignment clearly demonstrates a positive size‐assortative mating pattern, where females have a strong disposition to mate with comparatively larger males. Moreover, we show that this within‐pair size difference was larger in the reserve than in the fished area. We also documented that male size (body and claws) strongly influenced their mating success inside the reserve, while selection differentials on these traits were weaker and not significant in the fished area. Sexual selection was stronger on relative claw size, rather than on absolute claw and body size. Overall, our findings suggest that fishing can greatly affect mating patterns, with potential consequences for reproductive output and the rate and trajectory of fisheries‐induced evolution.

### 4.1 The effect of fishing on mating patterns

Having been afforded protection from fishing for almost a decade, lobsters in the reserve might display a good depiction of what can be considered more “natural” mating behaviour, given that females have access to a wider diversity of male phenotypes. Therefore, the increased scope for sexual selection on male traits is the likely explanation for the higher positive selection differentials in the marine reserve relative to the fished area. Although females in the fished area tended to mate with males larger than themselves, the average difference in body size between sexes was much smaller (♂ > ♀; 6.4%) than in the reserve (♂ > ♀; 22.5%). These results are in line with those for wild‐mated female American lobsters obtained by Gosselin, Sainte‐Marie, and Bernatchez (2003), who found a positive size‐assortative mating pattern in larger females caught in an area of moderate fishing pressure, but a random mating pattern in a site more heavily fished. In the fished area, the lower density of lobsters and the fact that females were about the same size as males in this area imply that females would have more difficulties finding a larger mate. When individuals are more sparsely distributed, sexual selection is likely to be relaxed through lower encounter rates between mates and competitors behaviour(Arnqvist, 1992; Conner, 1989). Further, high fishing mortality of large lobsters should free up more good shelters than would typically be available to smaller males, whose occupancy of better shelters ought to increase their chances with females (Atema, 1986; Cowan & Atema, 1990; Debuse, Addison, & Reynolds, 1999, 2003).

High fishing mortality could also explain the lower assignment probability for males in the fished area in 2010, as a male should have lower chances of surviving and successfully mate the following year, relative to a similar sized male in the reserve. This implies that the selection differentials may not be purely due to sexual selection but may also reflect an unidentified component of fisheries selection against large individuals. Arguably, results from this work show that selective harvesting is indeed affecting mating patterns, but we only compare the trait distributions of males with and without paternity on the broods (females) sampled. The consequences for true fitness and selection can therefore only be inferred. However, we find no reason to assume different trait distributions between the observed and unobserved fathers.

Contrasting no‐take reserves and complementary monitored control areas where harvesting continues as usual may be one of the best options available to study the effects of harvesting regimes in situ. To test the generality of the findings, and indisputably attribute spatial variability in ecology to that in fishing pressure, the approach should be tested using multiple pairs of reserves and fished areas. Moreover, temporal replicates, tracking several selection episodes and ideally also including natural and fisheries selection, could be used to estimate life time fitness and to test individual consistency and temporal stability in sexual selection.

### 4.2 Drivers of sexual selection in clawed lobsters

Female choice appears to play an important role in driving the positive assortative mating pattern; some of the largest males had mated with small females, while the largest females never mated with small males. Females can have a direct benefit from choosing larger males. First, female clawed lobsters usually moult in a males’ shelter, where she will mate soon after and cohabitate for some time, a strategy believed to increase successful pre‐ and postcopulatory guarding of the soft‐shelled female (Atema, Jacobson, Karnofsky, Oleszko‐Szuts, & Stein, 1979; Cowan & Atema, 1990; Karnofsky & Price, 1989; Karnofsky et al., 1989a,b). Secondly, large male decapods have greater sperm reserves, are capable of tailoring ejaculate load to the size of the female and replenish depleted sperm faster than smaller males (Gosselin et al., 2003; Jivoff, 1997; Kendall, Wolcott, Wolcott, & Hines, 2001; MacDiarmid et al., 1999). Thus, the narrow time window of receptiveness to mate, the need for protection during moulting and sperm quantity are plausible reasons for females to choose larger males. On the other hand, males could be reluctant to mate with smaller females if this comes at the cost of lower mating opportunities with a larger, more fecund female due to the time‐out period of the mating event. Nevertheless, it is reasonable to assume that male lobsters are less choosy than females, since an intermoult male can produce sperm all‐year‐round and is able to inseminate multiple females within a breeding season (Waddy et al., 2017).

The univariate selection gradients (mean standardized) on both claw (3.04) and body size (3.56) were relatively high, both being well above the median (1.93) calculated from 140 published estimates (Hereford et al., 2004). Interestingly, relative claw size appears to be the trait driving sexual selection in male lobsters, along with weaker selection on body size according to the most parsimonious multivariate model. Sexually selected structures like claws, with dual function of combat and display, are likely to be honest signals of male quality to competitors and choosy females (Berglund, Bisazza, & Pilastro, 1996; Grafen, 1990). Relative claw size might therefore be a better measure of male quality than absolute claw and body size, which could simply be due to chance survival to old age. Fitness benefits accruing to large males with relatively large claws are well documented in Fiddler crabs, where large‐clawed males win more competitions and attract more females than small‐clawed males (Christy, 1983; Oliveira & Custodio, 1998; Pratt & McLain, 2002). In both European and American lobster, larger claws are found to increase male competitive abilities and to be a better predictor of victors than body size (Atema & Cobb, 1980; Elner & Campbell, 1981; Van Der Meeren & Uksnøy, 2000). Note that we did not find support for stabilizing selection on body and claw size, implying that also very large individuals maintain high male–male competitiveness and/or female attraction.

### 4.3 Multiple matings and sperm limitation

Single paternity on female broods was the prevalent fertilization pattern, but two females caught in the fished area had evidence of being sired by two different males (2 out of 97 broods analysed). In contrast, a recent study in a region of the United Kingdom found no incidence of multiple paternity in the European lobster (Ellis et al., 2015). Both cases found in our study had contributions highly skewed in favour of a primary male and only a single offspring from each brood deviated from the other nine siblings. Multiply‐sired crustacean broods have often shown to have high level of paternal skew (e.g., Bailie, Hynes, & Prodöhl, 2011; Streiff, Mira, Castro, & Cancela, 2004; Yue et al., 2010). However, because of our method with only a limited sample of offspring, we did not have statistical power to detect a secondary parental sire of 9:1 skew with high probability. Thus, besides the two cases that were discovered by chance, it is possible that additional multiple sired broods were present among our single sired broods but went undetected.

Multiple paternal fertilizations have been documented in American lobster populations and linked to sperm limitation due to fisheries‐induced sex ratio imbalance (Gosselin, Sainte‐Marie, & Bernatchez, 2005). Whether it is cause for concern for our European lobster remain unknown, but the finding that females mate with relatively smaller males (presumably with lower sperm storages) in the fished area indicates that the likelihood of sperm limitation is present. As for males, we found eight individuals with known identities that had mated with more than one sampled female, but they did not differ in size from those with a single mating. Seven of these males came from the reserve, where the higher population density suggests increased opportunities for males to monopolize and mate with multiple females (Kokko & Rankin, 2006; Shuster & Wade, 2003).

### 4.4 Implications for fisheries‐induced evolution and management perspectives

When mating is nonrandom for traits under opposing harvest selection (e.g., when larger males are both preferred by females and targeted in fisheries), a reduction in mean and variability in these traits due to fishing is expected to lead to faster harvest‐induced evolution than under the assumption of random mating (Hutchings & Rowe, 2008). To our best knowledge, our study on European lobster provides the first empirical support for weakened sexual selection due to fishing. If fisheries continue to target individuals (particularly males) with higher relative reproductive success, the weakening of sexual selection will likely accelerate fisheries‐induced evolution towards smaller and less productive body size.

Despite the potential ramifications for rates of fisheries‐induced evolution, sexual selection tends to be left out of the equations in studies assessing this subject, with potential consequences for their conclusions (Hutchings & Rowe, 2008; Urbach & Cotton, 2008). The reason could be that obtaining data for estimating sexual selection is often more challenging than for natural and fisheries‐induced selection. In spite of this, we encourage inclusion of a sexual selection component in future studies of fisheries‐induced evolution because the genetic variation underlying sexually selected characters may be much higher than for nonsexually selected traits (Pomiankowski & Moller, 1995). Therefore, we may anticipate stronger evolutionary effects than on other phenotypic traits (Urbach & Cotton, 2008).

A general objective in an evolutionarily enlightened management framework should be to minimize harvest‐induced evolution and loss of adaptive potential in populations (Jørgensen et al., 2007). Accounting for evolutionary processes in management can potentially increase long‐term yield, the resilience to population collapse and ecosystem stability (Zimmermann & Jørgensen, 2017; Mollet, Poos, Dieckmann, & Rijnsdorp, 2016). Fishing that can maintain or increase the variability of sexually selected traits (that correlate genetically with body size) are predicted to slow evolution towards smaller body size relative to the scenario of random mating (Hutchings & Rowe, 2008; Uusi‐Heikkilä, Lindström, Parre, Arlinghaus, & Kuparinen, 2016). This may be achieved by changing the selectivity of fishing, such as restricting harvest of large individuals through gear modifications (e.g., reducing entrance diameter in traps) or maximum size limit/harvest slots (Hutchings and Fraser 2008, Zimmermann and Jørgensen 2017). A shift in management towards protection of large individuals can also restore age and size structure and balance sex ratios (Birkeland & Dayton, 2005; Tiainen, Olin, Lehtonen, Nyberg, & Ruuhijärvi, 2017; Halvorsen, Sørdalen, Durif, & Vøllestad, 2016), which should have positive effects on populations productivity and environmental resilience (Arlinghaus, Matsumura, & Dieckmann, 2010; Gwinn et al., 2015; Matsumura et al., 2011).

Long‐term overfishing has left the European lobster in Norway at a historically low level and profoundly diminished the prospects of individuals reaching a high age or large size. Thus, fisheries‐induced evolution may have already left considerable footprints. For the 2017 fishing season (starting 1 October), a maximum size limit of 320 mm total length (~116 mm CL) was implemented for lobster caught along the Skagerrak coast. As for the benefit of spatial management, a handful of small reserves established along the coastline are unlikely to have any strong effects on the evolutionary trajectory. If, however, the number and size of reserves are increased, with sexual selection recovering within, the potential for increased reproductive output from large females (mated with large males) and spill‐over of larger, more “attractive” males from the reserves could possibly strengthen sexual selection and buffer fisheries‐induced evolution in fished areas (see also: Baskett and Barnett 2015).

In conclusion, our paper presents novel empirical support for how fishing affects mating behaviour in wild European lobster. Selective fishing reduces the phenotypic variability for sexual selection to act upon, but at the same time, the strength of sexual selection may be relaxed through lowered density and biased sex ratio. Sexual selection is an integral part of evolution and should therefore be mandatory to consider in evolutionary enlightened management.

## CONFLICT OF INTEREST

None Declared.

## DATA ARCHIVING STATEMENT

Data available from the Dryad Digital Repository: https://doi.org/10.5061/dryad.1b1f023.
