# Supplementary figures and images for: Harvesting changes mating behaviour in European lobster
Source: Evol Appl. 2018 Mar 22;11(6):963–77. doi: 10.1111/eva.12611 (PMC5999211; doi:10.1111/eva.12611)

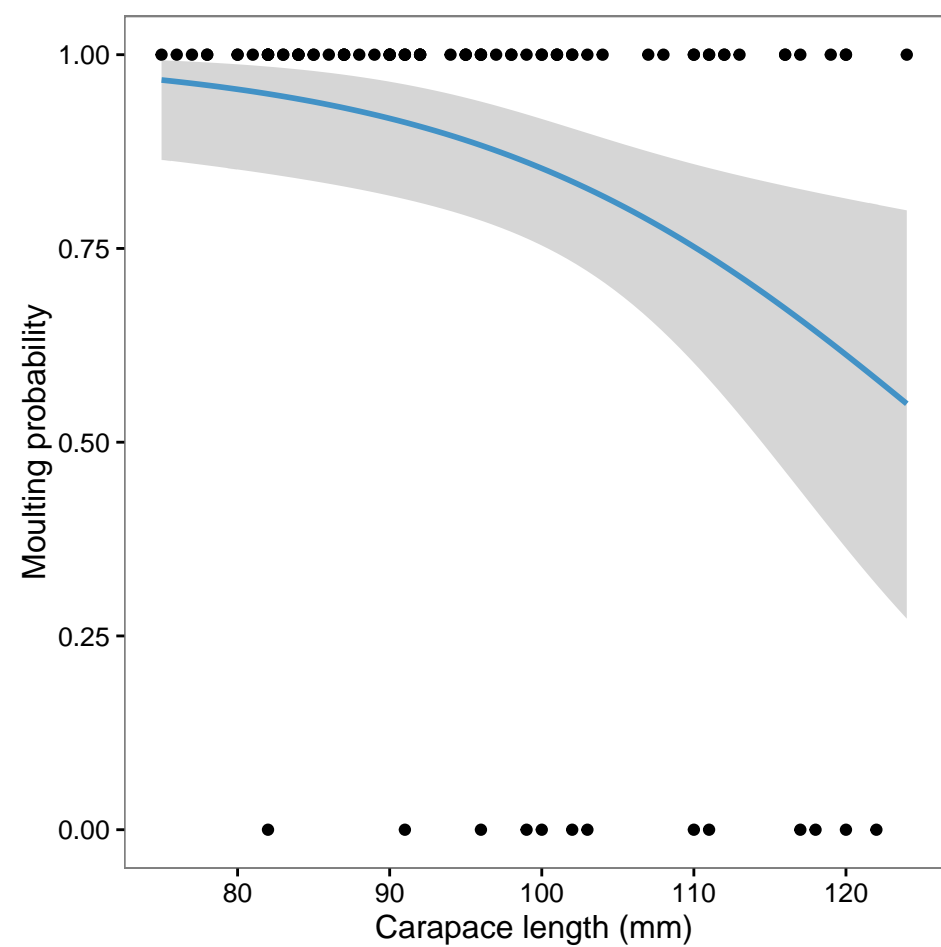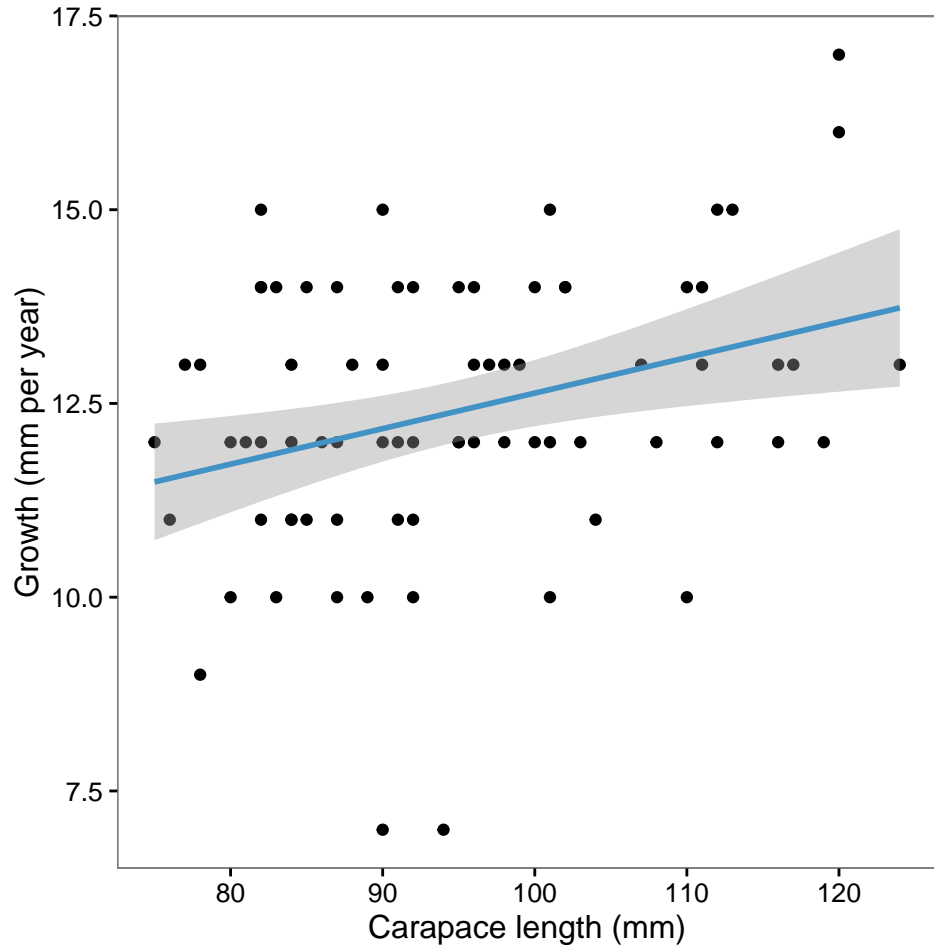

Supplement: Supplementary file 1 [file EVA-11-963-s001.pdf]

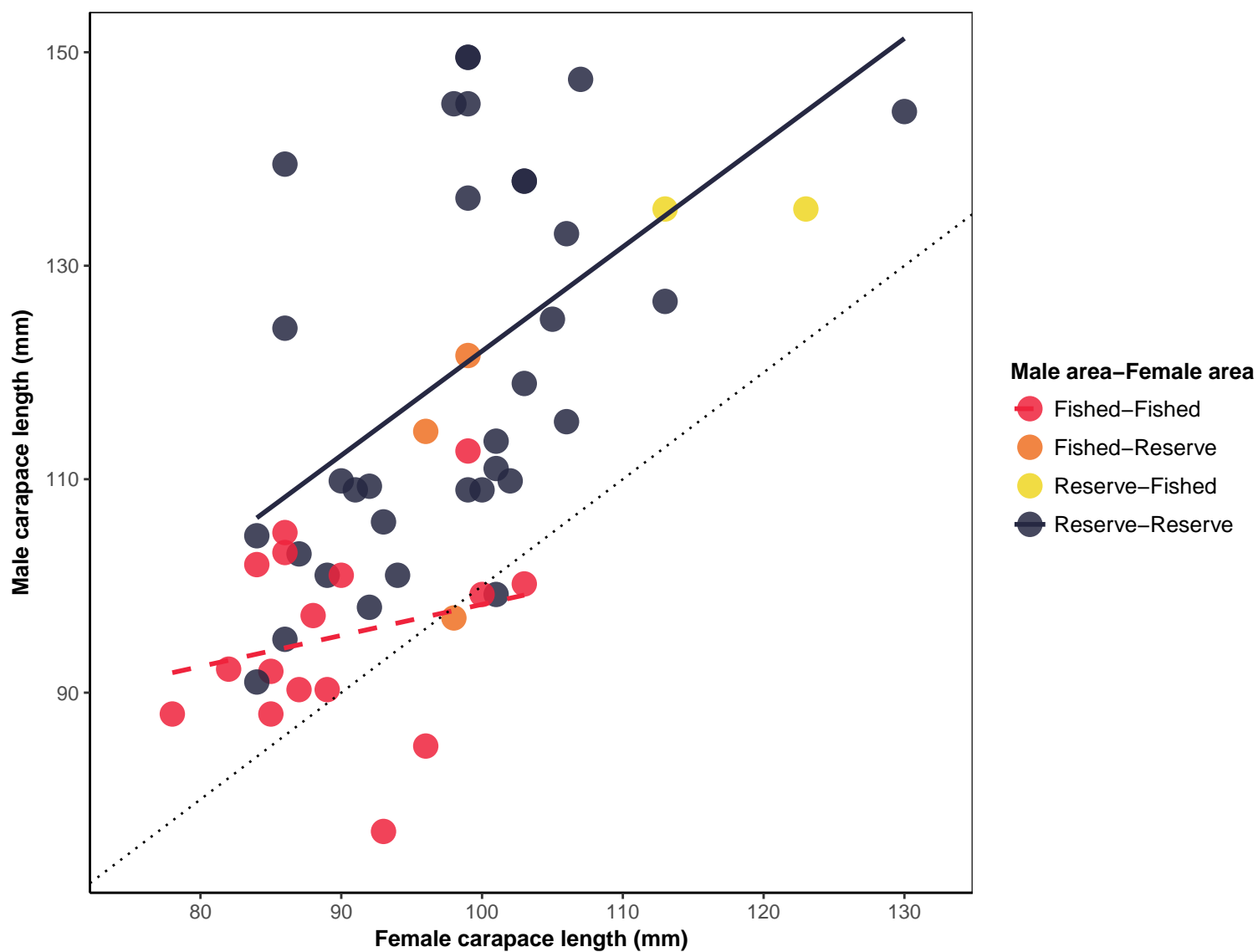

Supplement: Supplementary file 2 [file EVA-11-963-s002.pdf]
