# Supplementary material for: Harvesting changes mating behaviour in European lobster
Source: Evol Appl. 2018 Mar 22;11(6):963–77. doi: 10.1111/eva.12611 (PMC5999211; doi:10.1111/eva.12611)
Supplement: Supplementary file 3 [file EVA-11-963-s003.docx]

**Supplementary information S1: methods and results**

**S1.1 DNA extraction and genotyping**

The DNA was extracted with E.Z.N.A. Tissue DNA Isolation kit (Omega Bio-Tec inc.) using columns. The procedure followed the manufacturers guidelines, only deviating by preparing HiBind DNA mini columns with equilibration buffer (100 µl) and ddH_2_O (100 µl) separately. One offspring sampled from each pleopod was chosen (a total of 10 per female), crushed, and placed in a separately labelled tube. Pleopod tissue sampled from the female and male was cut into pieces of 30 mg before being digested overnight. To mitigate the low DNA concentration in some of the offspring samples, we eluted each sample in 50 µl buffer.

We carried out PCR amplifications on MyCycler ^™^ (Bio-Rad) using fluorescent- dyed forward primers (Life Technologies). One microliter of DNA was used for the PCR amplification, for a 10-μL total reaction volume, in one triplex (loci *hgd106, hgd111* and *hgc118*), three duplexes (loci *hgc111* and *hgc131, hgc129* and *hga8, hgb4* and *hgb6*) and one simplex (locus *hgc120*). Cycling condition for the triplex and duplex (*hgc111* and *hgc131*) included an initial 5-min phase of denaturation at 95 ̊C, followed by 35 cycles of 95, 56, and 72 ̊C for 30, 60, and 60 s, respectively, ending with a final elongation step at 72 ̊C for 15 min. Cycling condition for the simplex deviated by reducing the primer annealing and sequence extension to 30 s, while for the two duplexes (*hgc129* and *hga8, hgb4* and *hgb6*) cycling number was increased to 40.

Fragment analysis of PCR products was carried out on capillary sequencers CEQ^™^8000 (Beckman Coulter) and ABI ^™^ 3130xl (Applied Biosystems). Loci *hgd106, hgd111*, *hgc118* and half of samples with loci *hgc111,* *hgc131,* and *hgc120* were separated on CEQ8000, the other half along with all 2013 samples for loci *hgd106, hgd111*, *hgc118* and all of *hgc129,* *hga8, hgb4* and *hgb6* were separated on ABI3130xl. For samples run on CEQ^™^8000, total volume per well was 38 µl including 0.5 µl size standard and 3 – 4 µl PCR product mixture, where the duplex and simplex were combined in a pool-plex. Genotypes were scored manually using ceq^™^ 8000 genetic analysis system v 8.0. For samples run on ABI ^™^ 3130xl, PCR products were diluted 1:10 with ddH_2_O and pooled with 0.8 µl (*hgc129,* *hga8)* and 0.6 µl (*hgb4,* *hgb6)* respectively. Samples were then prepared by adding 0.15 µl of -250 LIZ (Applied Biosystems) internal size standard and 8.33 µl of formamide Hi-Di sample loading solution (Applied Biosystems) to 2 µl of the diluted PCR mixtures.

**S1.2 Genetic analysis and genotyping errors**

Genotypes were manually scored for each individual based on characteristic peaks using genemapper v3.7 (Applied Biosystems). As the length of the alleles slightly differed between the instruments, msatallele (Alberto, 2009), a script build on R, was used to bin the scored raw sizes from both fragment analysers and correctly calibrate the results from the two. All females and ~30% of males and eggs were screened on both sequencers for calibration. The script was modified to 2, 3 and 4 base pair repeats to suit the loci specifications.

Locus-specific genotyping error rates were estimated with a combination of methods. cervus was used to compare observed heterozygosity (*H*_O_) and expected heterozygosity (*H*_E_), and to identify any mother-offspring mismatches. We then used maximum likelihood (ML) programs pedant v. 1.0 (Johnson & Haydon, 2007) and micro-checker v. 2.2.1 (Van Oosterhout *et al*., 2004) to model approximations of allelic drop-out (ε_1_) and false allele (ε_2_) error rates, with two different sample sets. First, pedant was used to estimate locus-specific ε_1_ and ε_2_ separately by using repeat genotypes. Depending on the locus, ~7-8% of all adult and offspring samples were genotyped twice and used as replicates. Second, to better account for the presence of null alleles in the drop-out model, which also causes a homozygote excess similar to allelic drop-out, the frequency of null alleles was assessed with micro-checker based on all adult samples. When implementing the locus specific error rates (ε_1_ and ε_2_) for any given loci in the paternity analysis, we used micro-checker’s estimates as the ε_1_ error rate when micro-checker returned higher estimates than pedant’s drop-out model. This is because pedant is based on a subset of samples while micro-checker is based on all adult genotypes. Further, if the model of ε_2_ error rate in pedant returned zero for any loci, a conservative non-zero value of 0.01 was specified to allow some genotypic mismatches in the parentage analysis (Wang, 2004) (see **Table 1** for error rates).

**S1.3 Paternity analysis**

We assigned parentage using colony v 2.0 (Jones & Wang, 2010a; Wang, 2004), a full-pedigree likelihood program (Markov-chain Monte Carlo method) that provides the most probable configuration in assigning sib-ship and parentage among individuals. All individuals are divided into subsamples of offspring, mothers and fathers from which individuals are assigned to various numbers of family clusters. The algorithm calculates the likelihood of one pedigree cluster and compares the likelihood to other possible pedigrees to identify the most parsimonious cluster. Besides assigning parentage based on candidate parents, colony also reconstructs genotypes for the missing parent. Sampling a wild population, we expect to have incomplete representation of true fathers in the analysis. The assumed proportion of true fathers among the paternal candidates sampled was set to 50% based on results from preliminary runs in colony and cervus where we tested how various proportions affected the assignment rate. With very little variation in assignment results, colony showed robustness in handling uncertainty in the sampling rate, which is also supported elsewhere (Jones & Wang, 2010b; J Wang & Santure, 2009). colony allows both females and males to be polygamous, which is a prerequisite for testing multiple paternities in regard to both sexes. We chose to let colony update allele frequencies during assignment runs, since the total number of male contributions was inflated when allele frequencies were fixed. We accepted paternities assigned with 95% confidence or higher. This helped minimize false positive and false negative assignments and avoided overestimating the level of multiple paternity in the population. The best configuration given by colony was used to determine the number of males fathering the offspring. Although not all fathers were sampled, colony can infer their genotypes from the pedigree analysis to the number of mates to each female. The input files were set up with two replicate runs and analysed with the highest precision settings with full-likelihood, and with very long runtime on a PowerEdge M820, Linux CentOS 6.7 machine. No parentage was assigned to males sampled in 2013, so all males from this year were removed from further analysis, reducing the number of males to 563.

**S1.4 Population descriptive**

Significant linkage disequilibrium was detected in three out of 45 pair comparisons (6.6%) after accounting for multiple testing. Though colony does not implicitly account for linkage between loci, low levels of linkage disequilibrium are unlikely to have a large effect on the outcome of parentage analysis give the size of the sampled population (Amos *et al*.,1992; J Wang & Santure, 2009). Disequilibrium has not been found in a more wide-ranging population of European lobster in Norway (André & Knutsen, 2009), south-western UK (Ellis *et al*., 2015) or in the Irish sea (latter lost significance after Bonferroni correction) (Watson *et al*., 2016). Higher level of affinity between lobsters within our smaller study system would be more expected than within samples gathered across larger areas. Five loci (*C120, A8, B4, B6* and *C129*) did not conform to HWE after accounting for multiple testing (P < 0.05). Three of the loci (*A8, B6* and *C129*) also showed potential evidence of null alleles at frequencies 0.040 – 0.062, which could partially explain their deviation from HWE and cases of mismatches between maternal and offspring genotypes. Null-alleles at loci *A8* and *C129* were also found in Great Britain (Ellis *et al*., 2015). Systematic genotyping errors and hidden genetic structure from sampling in a local area could also explain why five loci were out of Hardy-Weinberg equilibrium (Dakin & Avise, 2004), where none were found in the more wide-ranging population study of European lobster in Norway.

**Litterature cited**

Alberto, F. (2009). MsatAllele_1.0: An R package to visualize the binning of microsatellite alleles. *The Journal of Heredity*, *100*, 394–7.

Amos, W., Barrett, J. a, & Pemberton, J. M. (1992). DNA fingerprinting: parentage studies in natural populations and the importance of linkage analysis. *Proceedings of the Royal Society B: Biological Sciences*, *249*, 157–62.

André, C., & Knutsen, H. (2009). Development of twelve novel microsatellite loci in the European lobster (Homarus gammarus). *Conservation Genetics Resources*, *2*, 233–236.

Dakin, E. E., & Avise, J. C. (2004). Microsatellite null alleles in parentage analysis. *Heredity*, *93*, 504–509. Journal Article.

Ellis, C. D., Hodgson, D. J., André, C., Sørdalen, T. K., Knutsen, H., & Griffiths, A. G. F. (2015). Genotype reconstruction of paternity in European lobsters (Homarus gammarus). *PLoS ONE*, *10*, 1–14.

Johnson, P. C. D., & Haydon, D. T. (2007). Maximum-likelihood estimation of allelic dropout and false allele error rates from microsatellite genotypes in the absence of reference data. *Genetics*, *175*, 827–42.

Jones, O. R., & Wang, J. (2010a). COLONY: a program for parentage and sibship inference from multilocus genotype data. *Molecular Ecology Resources*, *10*, 551–5. Journal Article.

Jones, O. R., & Wang, J. (2010b). Molecular marker-based pedigrees for animal conservation biologists. *Animal Conservation*, *13*, 26–34.

Van Oosterhout, C., Hutchinson, W. F., Wills, D. P. M., & Shipley, P. (2004). Micro-Checker: Software for Identifying and Correcting Genotyping Errors in Microsatellite Data. *Molecular Ecology Notes*, *4*, 535–538. Journal Article.

Wang, J. (2004). Sibship reconstruction from genetic data with typing errors. *Genetics*, *166*, 1963–1979. Journal Article.

Wang, J., & Santure, A. W. (2009). Parentage and sibship inference from multilocus genotype data under polygamy. *Genetics*, *181*, 1579–94.

Watson, H. V., McKeown, N. J., Coscia, I., Wootton, E., & Ironside, J. E. (2016). Population genetic structure of the European lobster (Homarus gammarus) in the Irish Sea and implications for the effectiveness of the first British marine protected area. *Fisheries Research*, *183*, 287–293.

**Supplementary information S2: figures and table**

(All figures are stored as separate pdf).

Figure S1: Predictions of yearly size change for males. Model predictions for molting probability and growth increment per year for those who had molted (increased the length with more than 5 mm). The data are from male lobster captured in consecutive years in a mark-recapture survey conducted in the study area of Flødevigen, Norway, 2006-2016. The predictions are used in the calculation of adjusted male body size when males with mating success were sampled in a different year than the females.

**Figure S2: Size-assortative mating (all pairs).** The relationship between body size (carapace length) of male (corrected sizes, see Materials and methods) and female European lobster that formed pairs (n = 51) in fished (red) and reserve (dark grey) area, and inter-area pairs where the male was from fished area and female was from reserve area (orange), and visa-versa (yellow) in the four-year period. Male *CL* is adjusted according to the year of the mating event. Value 1.0 and black stippled line (isometry, Y = X) marks where females and males are equal in size.

**Table S1:** **List of 97 female European lobster analysed for paternity separated in area and year of sampling**. ID of female, number of offspring analysed per female, average number of loci typed for each females’ offspring, the ID of primary (#1) and, in two cases, the secondary (#2) male, fertilization skew of paternity (in proportion) in each egg batch as determined by colony, and as determined by manual inspection (given as SP = single paternity/ MP = multiple paternity). Letter (R = reserve/ F = fished) denotes which area the known males (n = 44) were sampled and absence of a letter denotes the genotype ID (n = 57) of the most likely father of the egg batches. (^m^) denotes males that have mated with more than one female and are duplicated in table.

| **Area/ year** | **Female ID** | **Offspring analysed** | **Average no, of loci per offspring** | **male #1** | **male #2** | **Fertilization skew, determined by Colony** | **SP by male #1, or MP, determined by manual inspection** |
| --- | --- | --- | --- | --- | --- | --- | --- |
| **Reserve 2011** | 507 | 10 | 9,6 | R-0153 |  | 100 | SP |
|  | 639 | 10 | 9,9 | R-0626^m^ |  | 100 | SP |
|  | 678 | 10 | 9,6 | R-0571^m^ |  | 100 | SP |
|  | 681 | 10 | 9,2 | 36^m^ |  | 100 | SP |
|  | 699 | 10 | 9,8 | R-0668^m^ |  | 100 | SP |
|  | 715 | 10 | 9,9 | R-0079^m^ |  | 100 | SP |
|  | 718 | 10 | 9 | R-0397 |  | 100 | SP |
|  | 725 | 10 | 8,8 | R-0066^m^ |  | 100 | SP |
|  | 728 | 10 | 9,8 | R-0510^m^ |  | 100 | SP |
|  | 736 | 10 | 10 | R-0537 |  | 100 | SP |
|  | 886 | 10 | 9,9 | R-0771 |  | 100 | SP |
|  | 921 | 10 | 10 | R-0668^m^ |  | 100 | SP |
|  | 922 | 10 | 10 | R-0760 |  | 100 | SP |
|  | 944 | 10 | 10 | R-1361^m^ |  | 100 | SP |
|  | 946 | 10 | 9,6 | 41 |  | 100 | SP |
|  | 949 | 10 | 9,7 | 13 |  | 100 | SP |
|  | 953 | 10 | 9,8 | 42 |  | 100 | SP |
|  | 966 | 10 | 9,1 | R-0586 |  | 100 | SP |
|  | 971 | 10 | 10 | R-0284 |  | 100 | SP |
|  | 984 | 10 | 9,8 | R-1618 |  | 100 | SP |
|  | 1017 | 10 | 9,6 | R-0066^m^ |  | 100 | SP |
|  | 1029 | 10 | 9,6 | 3^m^ |  | 100 | SP |
|  | 1046 | 10 | 9,4 | R-0668^m^ |  | 100 | SP |
|  | 1053 | 10 | 9,7 | 6 |  | 100 | SP |
|  | 1055 | 10 | 9,6 | R-0070^m^ |  | 100 | SP |
|  | 1056 | 10 | 9,7 | R-1680 |  | 100 | SP |
|  | 1128 | 10 | 9 | 11 |  | 100 | SP |
|  | 1130 | 10 | 10 | R-0211 |  | 100 | SP |
|  | 1131 | 10 | 9,8 | 12 |  | 100 | SP |
|  | 1133 | 10 | 9,8 | R-0626^m^ |  | 100 | SP |
|  | 1147 | 10 | 9,7 | R-1361^m^ |  | 100 | SP |
|  | 1171 | 10 | 9,9 | 14 |  | 100 | SP |
|  | 1172 | 10 | 9,7 | R-0408 |  | 100 | SP |
|  | 625 | 10 | 9,8 | R-0079^m^ |  | 90:10 | SP |
|  | 754 | 10 | 9,7 | F-0992^m^ |  | 90:10 | SP |
|  | 933 | 10 | 10 | F-1111 |  | 90:10 | SP |
|  | 1048 | 10 | 9,3 | 5 |  | 90:10 | SP |
|  | 580 | 10 | 9,5 | R-0410 |  | 70:30:00 | SP |
|  | 881 | 10 | 9,8 | R-0070^m^ |  | 70:30:00 | SP |
|  | 1034 | 10 | 8,3 | R-1154 |  | 70:30:00 | SP |
|  | 912 | 10 | 10 | 36^m^ |  | 50:50:00 | SP |
|  | 1139 | 10 | 9,5 | R-0375 |  | 80:10:10 | SP |
| **Fished 2011** | 806 | 10 | 9,9 | F-0455^m^ |  | 100 | SP |
|  | 807 | 10 | 9,6 | 37 |  | 100 | SP |
|  | 819 | 10 | 9,7 | 38 |  | 100 | SP |
|  | 820 | 10 | 10 | F-0108 |  | 100 | SP |
|  | 833 | 10 | 10 | 39 |  | 100 | SP |
|  | 839 | 10 | 9,9 | 33 |  | 100 | SP |
|  | 864 | 10 | 9,9 | 40 |  | 100 | SP |
|  | 1006 | 10 | 7,8 | 1 |  | 100 | SP |
|  | 1011 | 10 | 8,6 | 2 |  | 100 | SP |
|  | 1041 | 10 | 8,5 | F-0992^m^ |  | 100 | SP |
|  | 1065 | 10 | 9,5 | 7 |  | 100 | SP |
|  | 1071 | 9 | 8,3 | F-1371 |  | 100 | SP |
|  | 1082 | 10 | 7,6 | F-0705 |  | 100 | SP |
|  | 1095 | 10 | 7,6 | F-1093 |  | 100 | SP |
|  | 1103 | 10 | 9,3 | 8 |  | 100 | SP |
|  | 1106 | 10 | 9,8 | 9 |  | 100 | SP |
|  | 1118 | 10 | 9,9 | 10 |  | 100 | SP |
|  | 1122 | 10 | 9,8 | F-0538 |  | 100 | SP |
|  | 1174 | 10 | 9 | F-0795 |  | 100 | SP |
|  | 1175 | 10 | 8,8 | 15 |  | 100 | SP |
|  | 1176 | 10 | 9,6 | 16 |  | 100 | SP |
|  | 1178 | 10 | 10 | 18 |  | 100 | SP |
|  | 1180 | 10 | 9,7 | 20 |  | 100 | SP |
|  | 1038 | 10 | 5,7 | 4 |  | 90:10 | SP |
|  | 1177 | 10 | 9,4 | 17 | F-1296 | 90:10 | **MP** |
|  | 1179 | 10 | 9,8 | 19 |  | 90:10 | SP |
|  | 842 | 10 | 9,5 | F-0131 |  | 80:10:10 | SP |
| **Reserve 2012** | 1617 | 10 | 10 | R-0373 |  | 100 | SP |
|  | 1640 | 10 | 10 | 34 |  | 100 | SP |
|  | 1642 | 10 | 9,7 | F-0704 |  | 100 | SP |
|  | 1648 | 10 | 8,1 | 31 |  | 100 | SP |
|  | 1663 | 10 | 9,5 | R-0759 |  | 100 | SP |
|  | 1683 | 10 | 9 | R-0611 |  | 100 | SP |
|  | 1647 | 10 | 9,3 | 35 |  | 90:10 | SP |
|  | 1665 | 10 | 8,4 | R-0635 |  | 90:10 | SP |
|  | 1632 | 10 | 7,9 | R-0011 |  | 80:20:00 | SP |
| **Fished 2012** | 1208 | 10 | 8,4 | R-0510^m^ |  | 100 | SP |
|  | 1211 | 10 | 9,6 | F-1210 |  | 100 | SP |
|  | 1221 | 10 | 7,5 | F-1248 |  | 100 | SP |
|  | 1223 | 10 | 8 | 3^m^ |  | 100 | SP |
|  | 1236 | 10 | 9,6 | 23 |  | 100 | SP |
|  | 1294 | 10 | 10 | F-1350 |  | 100 | SP |
|  | 1311 | 10 | 9,9 | 29 |  | 100 | SP |
|  | 1331 | 10 | 10 | 30 |  | 100 | SP |
|  | 1348 | 10 | 9,9 | R-0510^m^ |  | 100 | SP |
|  | 1369 | 10 | 9,8 | 32 |  | 100 | SP |
|  | 1218 | 10 | 8,3 | F-0992^m^ |  | 100 | SP |
|  | 1222 | 10 | 8,5 | 21 |  | 90:10 | SP |
|  | 1230 | 10 | 9,8 | 22 |  | 90:10 | SP |
|  | 1291 | 10 | 10 | 24^m^ | 26 | 90:10 | **MP** |
|  | 1300 | 10 | 9,5 | 27 |  | 90:10 | SP |
|  | 1363 | 10 | 9,2 | F-0357 |  | 90:10 | SP |
|  | 1383 | 10 | 9,4 | 28 |  | 90:10 | SP |
|  | 1252 | 8 | 7,8 | 24^m^ |  | 88:12:00 | SP |
|  | 1255 | 10 | 8,8 | 25 |  | 60:30:10 | SP |
